# Dynamic mechanical response and a constitutive model of Fe-based high temperature alloy at high temperatures and strain rates

**DOI:** 10.1186/s40064-016-2169-6

**Published:** 2016-04-23

**Authors:** Xiang Su, Gang Wang, Jianfeng Li, Yiming Rong

**Affiliations:** Key Laboratory of High Efficiency and Clean Mechanical Manufacture (Shandong University), Ministry of Education, Shandong University, Jinan, 250001 China; Beijing Key Lab of Precision/Ultra-Precision Manufacturing Equipment and Control, Beijing, 100084 China; Mechanical and Energy Engineering Department, South University of Science and Technology of China, Shenzhen, 518055 China

**Keywords:** Dynamic mechanical response, Constitutive model, SHPB, Adiabatic temperature rise, Stainless steel

## Abstract

The effects of strain rate and temperature on the dynamic behavior of Fe-based high temperature alloy was studied. The strain rates were 0.001–12,000 s^−1^, at temperatures ranging from room temperature to 800 °C. A phenomenological constitutive model (Power-Law constitutive model) was proposed considering adiabatic temperature rise and accurate material thermal physical properties. During which, the effects of the specific heat capacity on the adiabatic temperature rise was studied. The constitutive model was verified to be accurate by comparison between predicted and experimental results.

## Background

Steel alloys are used in a wide range of structural, naval, nuclear, and aerospace applications (Zaera et al. 2012), which have been widely investigated in machining process. Martensitic stainless steels demonstrate a high capability of energy absorption, making it suitable for military applications, where impacts and explosions are often involved (Abed et al. 2014). When it comes to the manufacturing of steam turbine important components, the Fe-based high temperature alloy shows excellent properties under extreme working conditions. The performance of steel alloys under high temperature should be carefully considered during the design and manufacturing process.

High-speed cutting is used for manufacturing steam turbine rotor blades for ultra-supercritical unit. The high cutting temperature generated during the cutting evidently influences tool wear, tool life, surface integrity, and chip formation. The high temperature also leads to the thermal deformation of the cutting tool, which is considered as the major source of error in the machining process (Abukhshim et al. 2006; Takeuchi et al. 1982; List et al. 2012; Özel and Altan 2000).

In order to achieve desirable performance for high-speed cutting, it is essential to study the material dynamic behavior combined with the boundaries conditions (Hortig and Svendsen 2007). Therefore, investigations based on modeling and simulation of the process are essential (Merchant 1945). During the simulation of manufacturing, the constitutive model is the most important element that describes the performance of the material, which is presented as the flow stress. Flow stress, which is the resistance to plastic flow under deformation, is affected by various microscopic parameters such as strain, strain rate, temperature, and microstructure (Guo and Nemat-Nasser 2006). Further, the knowledge of material deformation behavior under dynamic loading is crucial to ensure design and manufacturing reliably and in the engineering applications. Nowadays, several researchers have presented several phenomenological and physically based constitutive models (Guo et al. 2012; Khan and Liu 2012). Abed (2010) proposed microstructure-based constitutive relation to define the thermo-visco-plastic behavior of ferrite steel. The temperature and strain rate-dependent flow stress was mainly attributed to dislocation motion and intersection, i.e., the dynamic strain aging effect was excluded. Gupta et al. (2013a, b) put forward four constitutive models of Austenitic Stainless Steel 316, including the Johnson–Cook (JC) model, the modified Zerilli–Armstrong (ZA) model, a modified Arrhenius type equation and the Artificial Neural Network model. Four available plasticity models were also investigated and compared by conducting experiments using two ferrite steels over a wide range of temperatures and strain rates (Abed and Makarem 2012). Among the various types of model, Johnson–Cook model, Khan–Huang–Liang model, Voce–Kocks model and Power-Law model are the most common phenomenological constitutive models (Samantaray et al. 2009; Lin and Chen 2011; Liang and Khan 1999; Khan and Liu 2012). The flow stress is expressed as a function of strain, strain rate and temperature, which is described in Eq. (1):

1 $$\sigma = f(\varepsilon_{s} ,\dot{\varepsilon }_{s} ,T,n_{1} ,n_{2} ,n_{3} \ldots )$$

where n_1_, n_2_, n_3_, … are material constants which are fitted by regression analysis of the experimental results. Compared with physically based constitutive models, the phenomenological constitutive models have less material constants and are easy to be calibrated. However, for finite element analysis (FEA) application, the empirical constitutive models (Johnson–Cook model and Power-Law model) are the most commonly used. In the past decades, researchers have created and applied many modified Johnson–Cook constitutive models while the Split Hopkinson Pressure Bar (SHPB) technique was widely used (Mirza et al. 2013; Kajberg and Wikman 2007; Wang et al. 2013). But some researchers pointed out that the work hardening is overestimated with the increasing strain, and the current deformation temperature is higher than the reference temperature (Hou and Wang 2010; He et al. 2013). Compared to Johnson–Cook constitutive model, the flow stress in the Power-Law constitutive model is featured in two aspects. Firstly, with the increase of the strain, the flow stress will not rise in exponential form. Secondly, the thermal softening item is expressed in polynomial function, making it more flexible to be used for any deformation temperature (Yu et al. 2014).

Power-Law model is controlled by the synergistic effects of temperature, strain and strain rate. During the last decades, many researchers used the experimental stress–strain data from isothermal hot compression tests over a wide range of temperatures (1073–1473 K), but comparatively low strain rates (0.001–1 s^−1^) (Samantaray et al. 2009). Some researchers conducted quasi-static and high strain rate (up to 4500 s^−1^) experiments, whereas the high temperature environment was not considered (Pereira et al. 2001). Recently, a few investigations have been done to study the modeling process fully considering a wide range of strain rates and temperatures (Hor et al. 2013a, b; Lee et al. 2011; Hernandez et al. 2011; Wang et al. 2009).

**Fig. 1:**
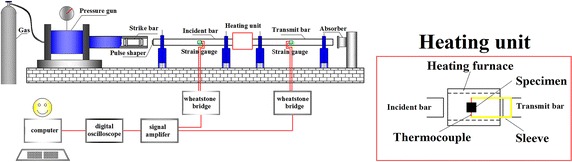
Schematic illustration of modified Split Hopkinson Pressure Bar (Yu et al. 2014)

Above all, as the SHPB compressive loading experiment is an adiabatic deformation process, the adiabatic temperature rise should be carefully considered. In the following study, the dynamic mechanical response of martensitic stainless steels is investigated using the Split Hopkinson Pressure Bar with synchronically assembled heating system, as is shown in Fig. 1. The temperatures ranges from 20 to 800 °C and strain rates are in the range of 3000–12,000 s^−1^. The comparison test of a quasi-static compressive experiment is carried out with servo hydraulic testing machine. The influences of strain rate and deformation temperature on the stress flow are evaluated according to the experimental data. The adiabatic temperature rises and accurate material thermal physical properties are taken into account to fit a phenomenological constitutive model. The accuracy of the constitutive model is verified through comparison with the experimental results.

## Experiment

### Materials

The Fe-based high temperature alloy used in this study belongs to the series of Cr12 % martensitic stainless steel, and it is used to manufacture steam turbine rotor blades of ultra-supercritical unit. In the experiments, the specimens were rolled after tempered heat treatment. The elementary composition (wt%) is shown in Table 1. The chemical composition of the Fe-based high temperature alloy used is complex, and it performs quite well. The basic mechanical properties determined under room temperature and static condition are shown in Table 2.

**Table 1:** Chemical composition of the Fe-based high temperature alloy (wt%)

Composition	C	Si	Mn	S	P	Cr	Mo	V
Content	0.08–0.1	≤0.10	0.35	≤0.010	≤0.015	10.0–12.0	0.10–0.40	0.15–0.25

Composition	Ni	Co	W	B	Al	N	Nb	Fe
Content	0.3–0.7	2.5–3.5	2.40	0.01–0.04	≤0.015	0.010–0.035	0.05–0.12	Bal.

**Table 2:** Basic mechanical properties of the Fe-based high temperature alloy

Mechanical properties	Tensile yield stress (MPa)	Tensile strength (MPa)	Elongation	Reduction of area	Rockwell hardness (HB)	Charpy V impact energy (J)
Content	≥620	≥885	≥0.15	≥0.45	≤321	≥10.7

### Quasi-static loading experiments

**Fig. 2:**
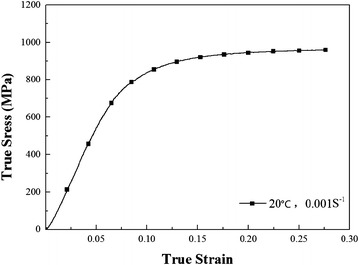
True stress–true strain curve at room temperature with strain rate 0.001 s^−1^

The quasi-static compression experiment at room temperature with strain rate 0.001 s^−1^ was performed by servo hydraulic testing machine in order to make a comparison. The true stress–true strain curve is presented in Fig. 2.

### Dynamic loading experiments

The dynamic compression experiments were performed by the modified Split Hopkinson Pressure Bar with synchronically assembled heating system. The experimental parameters variables were the temperatures and the strain rates: experiments with strain rate of 9000 s^−1^ at temperatures ranging from 20 to 800 °C, and experiments with strain rates ranging from 4000 to 12,000 s^−1^ at room temperature.

**Fig. 3:**
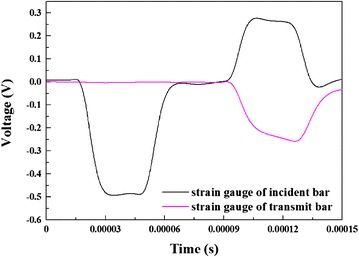
Typical voltage signals in the strain gauges (temperature: 20 °C, strain rate: 6000 s^−1^)

The signals (as shown in Fig. 3) received by strain gauges were firstly recorded by a Nicolet digital oscilloscope, and then these recorded signals were transferred into a computer for further data processing.

The average stress, strain and strain rate in the specimens can be calculated by the strain measured on the pressure bars according to following equations (Lindholm 1964):

2 $$\sigma_{e} = \frac{{AE\varepsilon_{T} }}{{A_{s} }}$$

3 $$\dot{\varepsilon }_{e} = \frac{{2C_{0} \varepsilon_{R} }}{{L_{s} }}$$

4 $$\varepsilon_{e} = \int\limits_{0}^{t} {\frac{{2C_{0} \varepsilon_{R} }}{{L_{s} }}dt}$$

where *E* is the Young’s modulus, *C*_0_ is the wave velocity, *A* is the cross-sectional area of the elastic bars respectively. *A*_s_ is cross-sectional area, *L*_s_ is the length of the cylindrical specimen respectively. *ɛ*_*T*_ and *ɛ*_*R*_ are elastic incident strain and elastic reflect strain caused by elastic incident stress pulse and elastic reflect stress pulse in elastic bar. *σ*_*e*_, *ɛ*_*e*_, $\dot{\varepsilon }_{e}$ are engineering stress, engineering strain and strain rate.

The true stress–true strain curves can be obtained from the following equations (Yu et al. 2014), of which the engine stress-engine strain curves were achieved from the experiments.

5 $$\varepsilon_{s} = - \ln (1 - \varepsilon_{e} )$$

6 $$\sigma_{s} = \sigma_{e} (1 - \varepsilon_{e} )$$

where *σ*_*e*_, *ɛ*_*e*_ are true stress and true strain.

Each experiment condition is performed at least three times in order to ensure the reliability of the experimental results. Only the results with good repeatability or less scatter can be accepted.

## Results and discussion

**Fig. 4:**
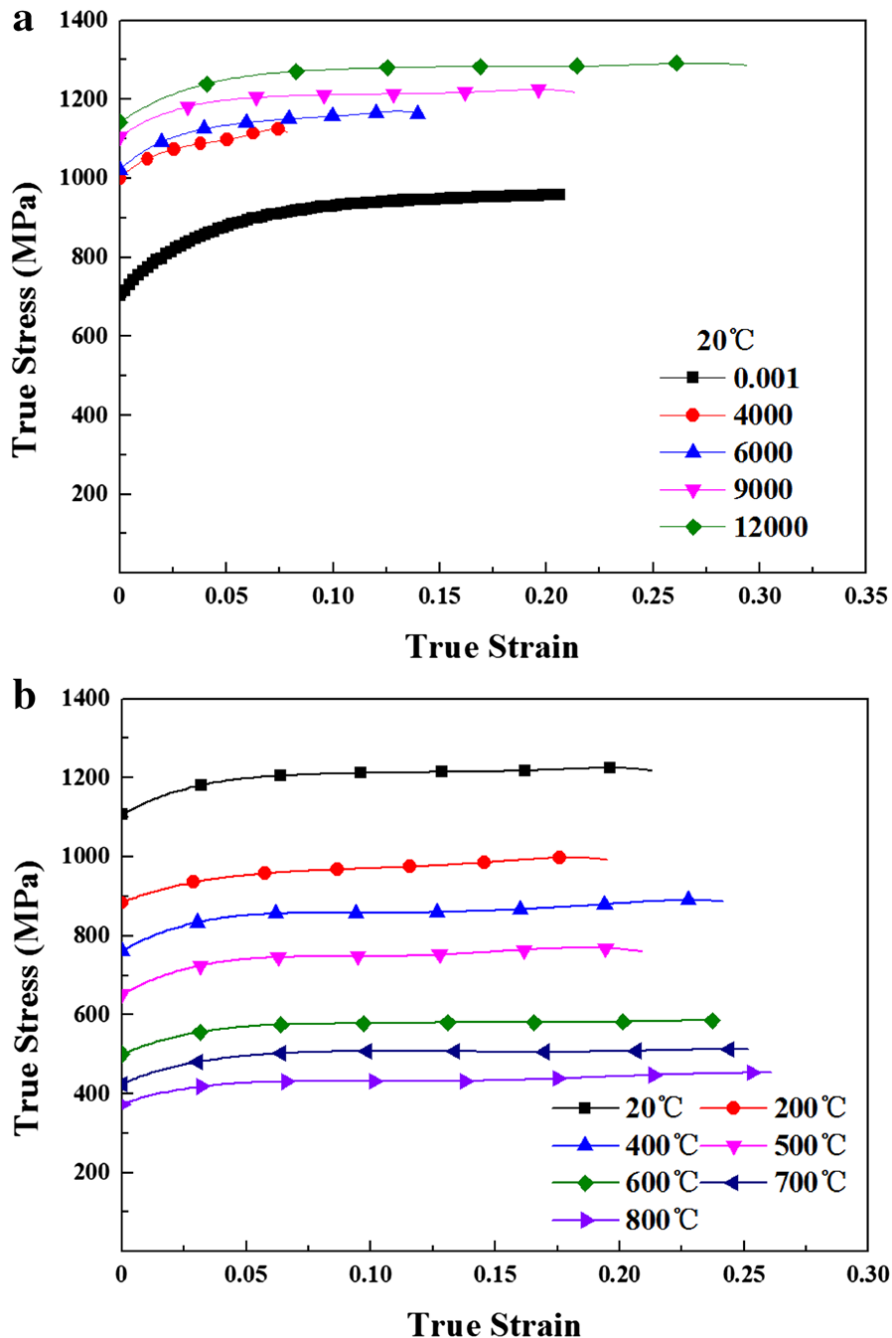
True stress–true strain curves: **a** different strain rates at 20 °C; **b** different temperatures with strain rate of 9000 s^−1^

Figure 4a shows the results of the experiments with strain rates ranging from 4000 to 12,000 s^−1^ at room temperature. The true strain-true stress curves with strain rate of 9000 s^−1^ at temperatures ranging from 20 to 800 °C are drawn in Fig. 4b.

**Fig. 5:**
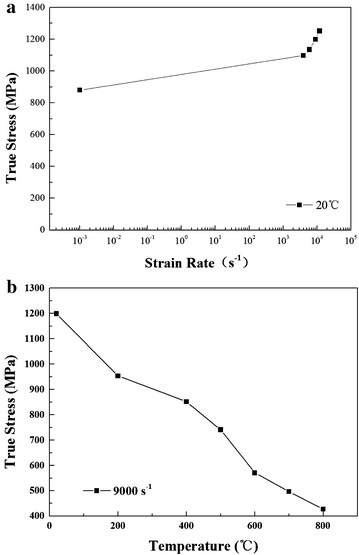
Variation of true stress: **a** effect of strain rate on the flow stress at 20 °C; **b** effect of temperature on the flow stress with strain rate of 9000 s^−1^

Figure 5a clearly depicts the relationship between flow stress and logarithmic strain rate when the true strain is 0.05 at room temperature. The relationship between flow stress and temperature with strain rate of 9000 s^−1^ at true strain (0.05) is shown in Fig. 5b. It can be concluded that both the strain rate and temperature have significant effects on the plastic flow behavior of the steel.

As is mentioned above, both temperature and strain have influence on flow stress. The temperature rises as the strain increases, which is regarded as adiabatic temperature (Khan et al. 2004). Therefore, accurate adiabatic temperature was taken into account during the fitting process of the constitutive model.

7 $$\Delta T = \frac{0.9}{{\rho_{s} C_{p} }}\int {\sigma_{s} d\varepsilon_{s} }$$

where Δ*T* is the adiabatic temperature, *ρ*_*s*_ is the density of specimen, *Cp* is the specific heat capacity of specimen.

**Fig. 6:**
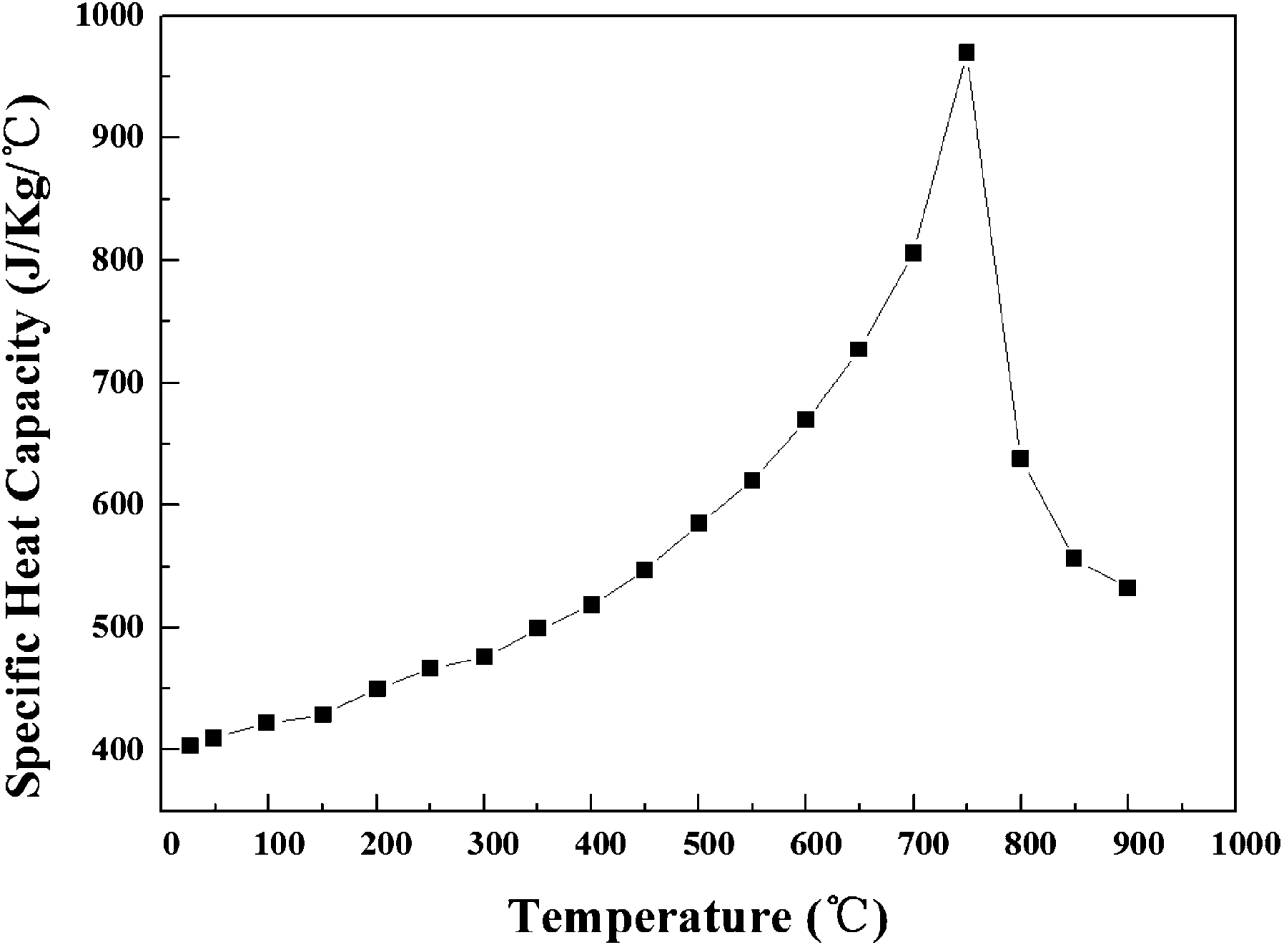
The specific heat capacity of material

The heat capacity variation which is represented as function of temperature was obtained through experiments. Figure 6 shows the experimental results, and the nonlinear fitting results are shown in Eq. (8).

8 $$\left\{ \begin{aligned} C_{p} = 0.4351 - 2.7840e^{ - 4} T + 1.1976e^{ - 6} T^{2} \quad 20\,^\circ {\text{C}} < T < 750\,^\circ {\text{C}} \hfill \\ C_{p} = 23.8935 - 0.05375T + 3.0899e^{ - 5} T^{2} \quad 750\,^\circ {\text{C}} < T < 900\,^\circ {\text{C}} \hfill \\ \end{aligned} \right.$$

As shown in Fig. 6, the specific heat capacity increases with temperature ranging from 20 to 700 °C, on the contrary while the temperature is above 700 °C.

**Fig. 7:**
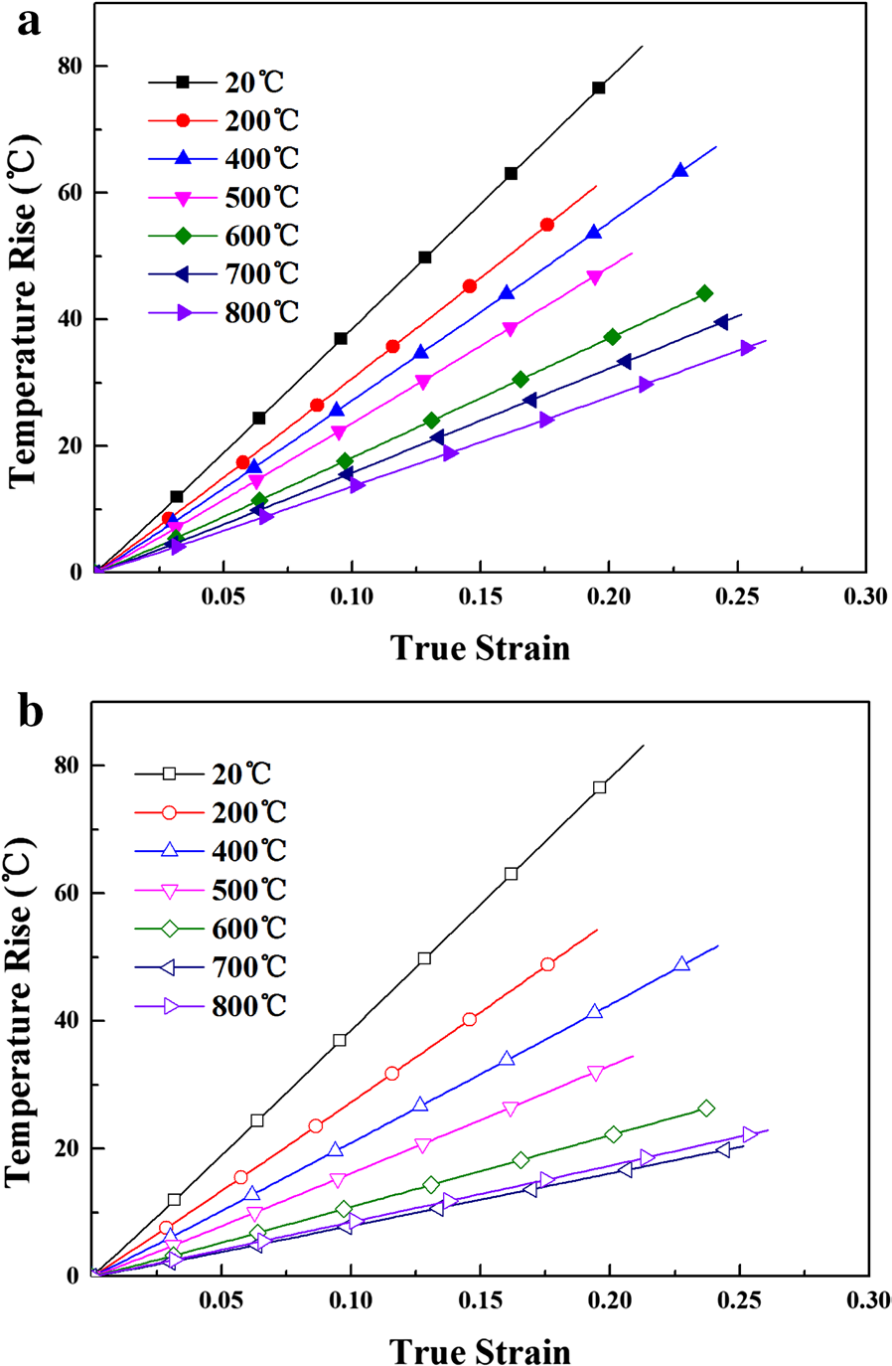
Temper rise with the strain rate of 8000 s^−1^ at various temperatures **a** specific heat capacity = 400; **b** specific heat capacity changed with temperature

In most study, the specific heat capacity was taken as a constant, but the specific heat capacity affect the adiabatic temperature rise obviously, especially at high temperature (200–800 °C) as shown in Fig. 7. The temperature rise calculated with specific heat capacity changed with temperature is much lower than that with the invariable specific heat capacity. So, in this study the temperature-depended specific heat capacity was used to guarantee the accuracy of the temperature rise.

As analyzed before, the flow stress decreases with the increase of temperature. However, the flow stress is balanced by work hardening and adiabatic thermal softening, which could be well exhibited in Power-Law model (Ranc et al. 2008).

## Constitutive model

In this section, phenomenological constitutive model of Power-Law relationship is proposed. The constitutive model is expressed as Eq. (9–13):

9 $$\sigma_{s} (\varepsilon_{s} ,\dot{\varepsilon }_{s} ,T) = g(\varepsilon_{s} ) \times \varGamma (\dot{\varepsilon }_{s} ) \times \varTheta (T)$$

10 $$g(\varepsilon_{s} ) = \sigma_{0} \left( {1 + \frac{{\varepsilon_{s} }}{{\varepsilon_{0} }}} \right)^{1/n}$$

11 $$\varGamma (\dot{\varepsilon }_{s} ) = \left( {1 + \frac{{\dot{\varepsilon }_{s} }}{{\dot{\varepsilon }_{0} }}} \right)^{1/m}$$

12 $$\varTheta (T) = c_{0} + c_{1} T + c_{2} T^{2} + c_{3} T^{3} + c_{4} T^{4} + c_{5} T^{5}$$

13 $$T = T_{ini} + \varDelta T$$

where *σ*_0_ is the yield stress at reference strain rate and the temperature, *T*_*ini*_ is the initial deformation temperature, *T* is the current deformation temperature, *ΔT* is the adiabatic temperature rise, *ɛ*_0_ is the reference strain, and $\dot{\varepsilon }_{0}$ is the reference strain rate. *m*, *n*, and *c*_*0*_ ~ *c*_*5*_ are material constants of constitutive model. In Eq. (9), the flow stress in the constitutive model is represented by the multiplication of three items (*g*(*ɛ*_*s*_), *Γ*($\dot{\varepsilon }_{s}$) and *Θ*(*T*)), which are used to describing the work-hardening effect, the strain-rate hardening effect and thermal softening effect, respectively. The three items are assumed to be independent.

### Determination of the constants

**Fig. 8:**
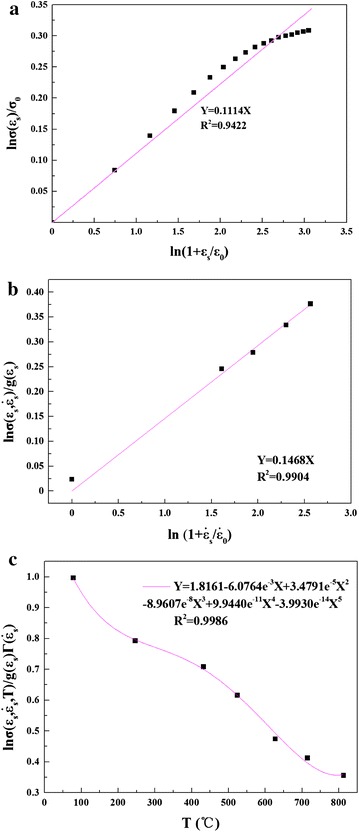
Fitting lines: **a** relationship between ln *σ*(*ɛ*_*s*_)/*σ*_0_ and ln (1 + *ɛ*_*s*_/*ɛ*_0_); **b** relationship between ${ \ln }\sigma \left( {\varepsilon_{s} ,\dot{\varepsilon }_{s} } \right)/g\left( {\varepsilon_{s} } \right)$ and $ln \left( {1 + \dot{\varepsilon }_{s} /\dot{\varepsilon }_{0} } \right)$; **c** relationship between $\sigma \left( {\varepsilon_{s} ,\dot{\varepsilon }_{s} , T} \right)/g\left( {\varepsilon_{s} } \right) \times \varGamma \left( {\dot{\varepsilon }_{s} } \right)$ and *T*

The true stress–true strain curves obtained from the compressive loading experiment under different strain rates and temperatures are used to determine the material constants of the constitutive model by regression analysis. Figure 8 shows the fitting lines, and the constants of the constitutive model are shown in Table 3.

**Table 3:** Material constants of constitutive model

*σ*_0_	*ɛ*_0_	*m*	$\dot{\varepsilon }_{0}$	*n*	*c*_0_	*c*_1_	*c*_2_	*c*_3_	*c*_4_	*c*_5_
702.8311	0.01	6.8120	1000	8.9767	1.8161	−6.0764e − 3	3.4791e − 5	−8.9607e − 8	9.9440e − 11	−3.9930e − 14

Above all, the Power-Law constitutive model can be expressed as follows:

14 $$\begin{aligned} \sigma (\varepsilon_{s} ,\dot{\varepsilon }_{s} ,T) & = 702.8311\left( {1 + \frac{{\varepsilon_{s} }}{0.01}} \right)^{1/8.9767} \times \left( {1 + \frac{{\dot{\varepsilon }_{s} }}{1000}} \right)^{1/6.8120} \\ & \quad \times (1.8161 - 6.0764e^{ - 3} T + 3.4791e^{ - 5} T^{2} \\ & \quad - 8.9607e^{ - 8} T^{3} + 9.9440e^{ - 11} T^{4} - 3.9930e^{ - 14} T^{5} ) \\ \end{aligned}$$

where *T* can be expressed in Eq. (15) considering the adiabatic temperature discussed in “Results and discussion” section.

15 $$\left\{ \begin{array}{ll} T = T_{ini} +\Delta T{\kern 1pt} & \quad T_{0} \le T < 800 \\ T = T_{ini} & \quad T \ge 800 \hfill \\ \end{array} \right.$$

### Verification of the constitutive model

**Fig. 9:**
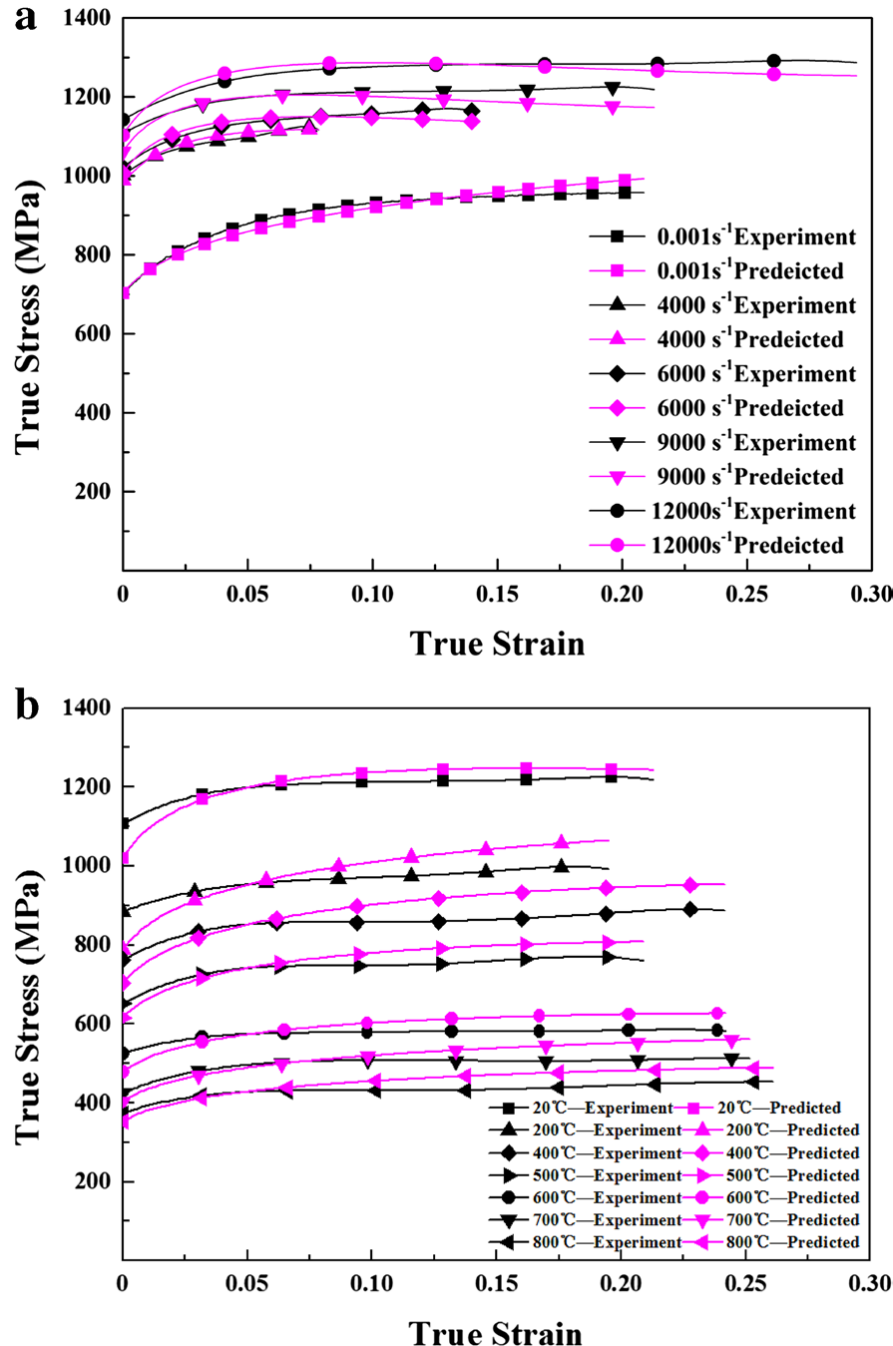
Comparison of flow stress measured in the experiment and predicted by the constitutive model **a** 20 °C, **b** 9000 s^−1^

The comparison of experimental flow stress and model predicted is shown in Fig. 9.

It can be seen from Fig. 9a that predicted results well correlate with the experimental results. The errors under high strain rates and high true stain are <5 %. The errors between predicted results and experimental results at strain rate 9000 s^−1^ with temperature ranging from room temperature to 800 °C are larger than that at room temperature with high strain rates, as is shown in Fig. 9b. But the errors are <10 %. So, the Power-Law constitutive model proposed in this study is accurate enough to describe the dynamic mechanical response of the steel under high strain rate and high temperature conditions.

## Numerical study of metal cutting process

In order to explain the practicability and accuracy, orthogonal metal cutting process was carried out by both experiments and the finite element method (FEM). The main cutting forces were taken as the research object.

### Orthogonal metal cutting experiment

The experiments of orthogonal cutting were performed on the DL-32M CNC lathe. The Kennametal grooving inserts (NG3125L-K313) with uncoated carbide was used in the experiments. All of the inserts were sharpened to meet the requirements listed in Table 4. In order to guarantee the radius of cutting edge, plenty of samples have been prepared. Then the equipment (GFM Mikro CAD) was applied to measure them, and the best ones were chosen as the samples for the experiments.

**Table 4:** Design of experiments by the orthogonal array

No.	Factors			
	V (m/min)	a_p_ (mm)	γ (°)	r (μm)
1	100	0.006	0	3
2	55	0.015	0	7
3	25	0.02	0	9
4	55	0.02	10	3
5	25	0.006	10	7
6	100	0.015	10	9
7	25	0.015	20	3
8	100	0.02	20	7
9	55	0.006	20	9

The work length of the insert is 1 mm in keeping with the settings of simulation. Kistler dynamometer typed 9257B was used to collect the cutting force. Nine groups of experiments were carried out, and the parameters are shown in Table 4. By this way, the workload was reduced and experimental effect was guaranteed.

### Numerical study of metal cutting process

The simulation of orthogonal metal cutting process was performed using commercial finite element software Third Wave AdvantEdge 6.4, which is widely adopted in the machining industry to facilitate the automation of production. The constitutive model obtained in “Constitutive model” section was input into the software as the costumer materials. The other thermal and mechanical properties of work piece are set as temperature-dependent thermal property. For example, Fig. 6 and Eq. (8) show the specific heat capacity of material. The thermal conductivity and thermal expansion are also temperature-dependent thermal properties, which were tested accurately. The Young’s modulus was set as 211 GPa, and the Poisson’s ratio was 0.33. The properties of cutting tool and coating were offered by the material library in the software. By some trial simulations, the friction coefficient between cutting tool and work piece was set as 0.5.

### Result and discussions

**Fig. 10:**
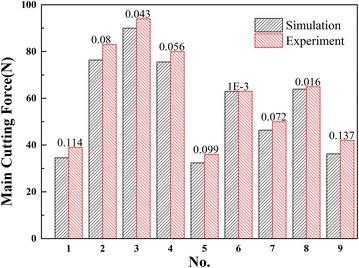
Comparison of the main cutting force between experimental and simulated results

For better comparison, the main cutting forces obtained from the experiments and simulations are listed in Fig. 10. It is obvious that the experimental and simulated results show the same tendency of changing. It is found that the cutting force increases with the increasing cutting depth, and meanwhile, the rake angle and the radius of cutting edge have significant influence on the cutting force.

The values of simulation results are smaller than the experiment ones. The average error of cutting forces is 7.86 %, which indicates good data reproducibility, and the error is clearly labeled in Fig. 10. The simulated results are consistent with the experimental results, confirming that the model of simulation can provide an accurate prediction of the main cutting force. In other words, the constitutive model obtained in this study is practicable and accurate.

## Conclusions

This paper investigated the dynamic mechanical response of the Fe-based high temperature alloy. The work hardening and temperature softening are significant at high strain rates and temperatures, and the results coincided with the actual machining process. The effects of the specific heat capacity on the adiabatic temperature rise was studied, it can be seen that the specific heat capacity affect the adiabatic temperature rise obviously, especially at high temperature (200–800 °C).

A Power-Law constitutive model considering accurate adiabatic temperature rise is obtained through regression analysis of the experimental data gathered from a modified Split Hopkinson Pressure Bar technique with synchronically assembled heating system. The constitutive model was verified to be accurate by comparison between predicted and experimental results. The main cutting forces obtained from the experiments and simulations were compared, of which the average error of cutting forces is 7.86 %, which indicates that the constitutive model obtained in this study is practicable and accurate.
